# Suppurative labyrinthitis associated with otitis media: 26 years’ experience^☆^^☆^

**DOI:** 10.1016/j.bjorl.2014.12.012

**Published:** 2015-12-11

**Authors:** André Souza de Albuquerque Maranhão, Valeria Romero Godofredo, Norma de Oliveira Penido

**Affiliations:** a Department of Otorhinolaryngology, Escola Paulista de Medicina, Universidade Federal de São Paulo (EPM-UNIFESP), São Paulo, SP, Brazil; b Department of Head and Neck Surgery, Escola Paulista de Medicina, Universidade Federal de São Paulo (EPM-UNIFESP), São Paulo, SP, Brazil; c Department of Medicine, Escola Paulista de Medicina, Universidade Federal de São Paulo (EPM-UNIFESP), São Paulo, SP, Brazil

**Keywords:** Otitis media, Labyrinthitis, Hearing loss, Otite média, Labirintite, Perda auditiva

## Abstract

**Introduction:**

Suppurative labyrinthitis continues to result in significant hearing impairment, despite scientific efforts to improve not only its diagnosis but also its treatment. The definitive diagnosis depends on imaging of the inner ear, but it is usually clinically presumed.

**Objective:**

To analyze the clinical factors and hearing outcomes in patients with labyrinthitis secondary to middle ear infections and to discuss findings based on imaging test results.

**Methods:**

Retrospective cohort study, based on the charts of patients admitted with middle ear infection-associated labyrinthitis.

**Results:**

We identified 14 patients, eight (57%) of whom were females and six (43%) males. Mean age was 40 years. Cholesteatomatous chronic otitis media was diagnosed in six patients (43%), acute suppurative otitis media in six (43%), and chronic otitis media without cholesteatoma was diagnosed in two patients (14%). Besides labyrinthitis, 24 concomitant complications were identified: six cases (25%) of labyrinthine fistula, five cases (21%) of meningitis, five cases (21%) of facial paralysis, five cases (21%) of mastoiditis, two cases (8%) of cerebellar abscess, and one case (4%) of temporal abscess. There was one death. Eight (57%) individuals became deaf, while six (43%) acquired mixed hearing loss.

**Conclusion:**

Suppurative labyrinthitis was often associated with other complications; MRI played a role in the definitive diagnosis in the acute phase; the hearing sequel of labyrinthitis was significant.

## Resumo

**Introdução:**

Labirintite permanece resultando em deficiência auditiva significativa, apesar dos esforços científicos para melhorar não só o diagnóstico, como também o tratamento. O diagnóstico definitivo é dependente de imagens da orelha interna, mas geralmente é presumido clinicamente.

**Objetivo:**

Analisar os fatores clínicos e os resultados auditivos em pacientes com labirintite secundária à otite média e discutir os achados dos exames de imagem.

**Método:**

Estudo de coorte retrospectivo, com base nos prontuários de pacientes diagnosticados com labirintite associada à infecção da orelha média.

**Resultados:**

Foram identificados 14 pacientes, oito (57%) do sexo feminino e seis (43%) masculino. Média etária de 40 anos. Otite média crônica colesteatomatosa foi diagnosticada em seis pacientes (43%), otite média aguda em seis pacientes (43%) e otite média crônica sem colesteatoma em dois pacientes (14%). Foram identificadas 24 complicações concomitantes: seis casos (25%) de fístula labiríntica, cinco casos (21%) de meningite, cinco (21%) de paralisia facial, cinco (21%) de mastoidite, dois casos (8%) de abscesso cerebelar e um caso (4%) de abcesso temporal. Houve uma morte. Oito (57%) indivíduos tornaram-se anacústicos, enquanto seis (43%) evoluíram para perda auditiva mista.

**Conclusão:**

Labirintite foi frequentemente associada a outras complicações; RNM auxiliou no diagnóstico definitivo da labirintite na sua fase aguda; a sequela auditiva da labirintite foi significativa.

## Introduction

The advent of antibiotics and immunizations in the last century led to a considerable decline in the incidence of complications from otitis media, and therefore a discussion on suppurative labyrinthitis associated with middle-ear infection may seem, at first glance, an outdated issue. However, complications still occur, particularly in developing countries, with significant morbidity (notably, hearing loss).^1^, ^2^, ^3^, ^4^, ^5^, ^6^ Leskinen et al. studied 50 patients treated for otitis media complications and, among the several complications listed in the study, suppurative labyrinthitis was considered the most disabling, in that all affected individuals developed profound or complete hearing loss.^2^

The diagnosis of suppurative labyrinthitis secondary to otitis media is essentially a clinical one, through the observation of vertigo, nystagmus, tinnitus and hearing impairment in the presence of a middle ear infection. In many diseases of the inner ear, the inflammatory process that ensues is usually presumed, rather than effectively diagnosed, and corticosteroids are empirically prescribed as the treatment of choice.^7^ The identification of suppurative labyrinthitis is usually more obvious, due to the magnitude and severity, of the symptoms, whereas in serous labyrinthitis symptoms are more subtle and many patients experience a satisfactory recovery with the treatment of underlying disorders of the middle ear.

The complex location of inner ear structures in the temporal bone, housed in the dense bone of the otic capsule, represents a significant barrier for the access and identification of any alterations in this region. Considering that the current knowledge about inner ear physiopathology is mainly derived from animal studies involving the collection of tissues and histological, molecular and inflammatory marker analyses, little is known about the mechanisms involved in diseases of the human inner ear *in vivo*.^8^

Imaging tests are important tools in an attempt to better understand the dynamics of inner ear inflammation. Currently the high-resolution computed tomography (HR-CT) best evaluates diseases affecting the bony labyrinth and nuclear magnetic resonance (NMR) imaging defines diseases that affect the inner ear and retrocochlear pathways. Recent advances in NMR techniques offer interesting opportunities for the study of cochlear structure, function and metabolism *in vivo*. The use of gadolinium as a contrast for the study of the inner ear adds sensitivity to the NMR, particularly for diseases such as labyrinthitis.^8^, ^9^, ^10^, ^11^

The aim of this research is to analyze clinical factors and hearing outcomes of patients with labyrinthitis secondary to middle ear infections and discuss the results of imaging tests.

## Methods

A retrospective cohort study was carried out based on medical records of patients treated at the Otology/Otoneurology department of a tertiary university hospital between 1987 and 2013. The study included patients whose medical records contained the diagnosis of labyrinthitis secondary to otitis media. Medical records with incomplete information were excluded, as well as those in which the diagnosis of suppurative labyrinthitis was not well established. The following data were collected from medical records: age, gender, medical history, type of middle ear infection, associated complications, audiometry and imaging test results. All patients received antibiotic therapy and systemic corticosteroids. The study was approved by the Ethics Committee of the institution under Protocol number 0081/10.

## Results

A total of 14 patients diagnosed with labyrinthitis associated with otitis media were identified. Eight were female (57%) and six (43%) were male. The mean age was 40, with an age range from 9 to 67 years (Table 1).

**Table 1:** Description of cases.

Case	Gender	Age	Otologic diagnosis	Comorbidity	Imaging test	Associated complication	Auditory outcome
1	F	59	CCOM	Diabetes mellitus	CT	Labyrinthine fistula	Severe MHL
2	F	20	NCCOM	AIDS	CT	Meningitis	Anacusis
3	M	9	CCOM	–	CT NMR	Labyrinthine fistula Meningitis Cerebellar abscess	Anacusis
4	M	39	CCOM	Chronic eosinophilic pneumonitis	CT	–	Anacusis
5	M	43	CCOM	–	CT	Labyrinthine fistula	Severe MHL
6	M	18	CCOM	–	CT NMR	Meningitis Labyrinthine fistula Temporal abscess	Anacusis
7	F	31	CCOM	–	CT	Meningitis Facial paralysis Labyrinthine fistula	Anacusis
8	F	17	CCOM	–	CT NMR	Meningitis Labyrinthine fistula Mastoiditis Cerebellar abscess	Anacusis
9	M	31	AOM	Wegener's Granulomatosis	CT NMR	Facial paralysis Mastoiditis	Moderate–severe MHL
10	F	52	AOM	Wegener's Granulomatosis	CT NMR	Facial paralysis Mastoiditis	Moderate–severe MHL
11	F	42	AOM	Wegener's Granulomatosis	CT NMR	Facial paralysis Mastoiditis	Moderate–severe MHL
12	F	64	AOM	Diabetes mellitus	CT NMR	Facial paralysis	Moderate MHL
13	F	41	AOM	–	CT NMR	Facial paralysis Meningitis	Anacusis
14	F	67	AOM	Diabetes mellitus	CT NMR	Mastoiditis	Anacusis

CCOM, cholesteatomatous chronic otitis media; NCCOM, non-cholesteatomatous chronic otitis media; AOM, acute otitis media; CT, computed tomography; NMR, nuclear magnetic resonance; AIDS, acquired immunodeficiency syndrome; MHL, mixed hearing loss.

**Figure 1:**
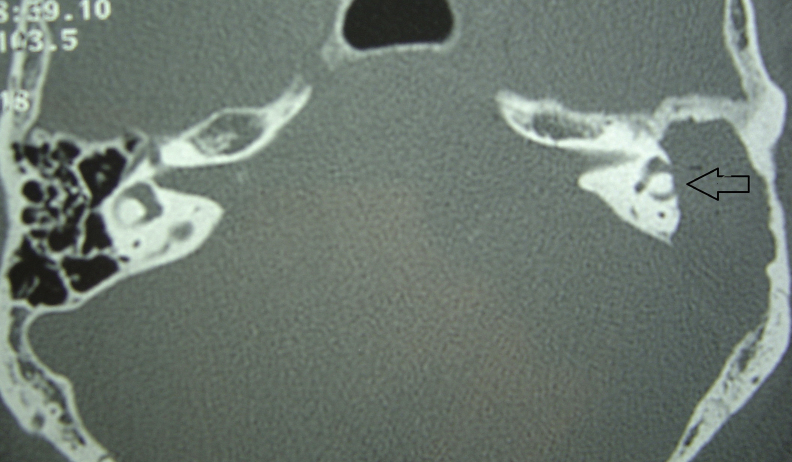
Temporal bones CT, axial view. The arrow shows erosion of the lateral semicircular canal on the left, in a patient with CCOM. *Note*: posterior erosion in the mastoid, adjacent to the sigmoid sinus.

**Figure 2:**
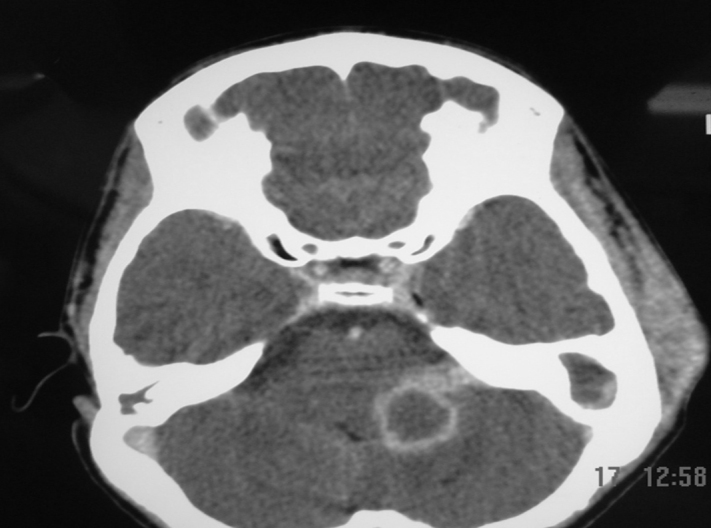
Brain CT with contrast, axial view and soft tissue window. It shows a cerebellar abscess on the left. *Note*: soft tissue edema, adjacent to the left temporal bone, consistent with mastoiditis.

All patients had otitis media before the diagnosis of labyrinthitis. Otitis media was classified as follows: cholesteatomatous chronic otitis media (CCOM) in six (43%) patients, acute otitis media (AOM) in six (43%) patients, and non-cholesteatomatous chronic otitis media (NCCOM) in two (14%) patients. Thirteen (93%) patients had one or more of the following associated complications (total of 24 recorded complications): labyrinthine fistula (Fig. 1) in six patients (25%, *n* = 24); meningitis, facial paralysis and mastoiditis in five patients each (21%, *n* = 24); cerebellar abscess (Fig. 2) in two patients (8%, *n* = 24), and temporal lobe abscess in one patient (4%, *n* = 24). There was one death in this study (case 7) (Table 2).

**Table 2:** Distribution of complications (*n* = 24).

Complication	*N*	%
Labyrinthine fistula	6	25
Meningitis	5	21
Facial paralysis	5	21
Mastoiditis	5	21
Cerebellar abscess	2	8
Temporal abscess	1	4

**Figure 3:**
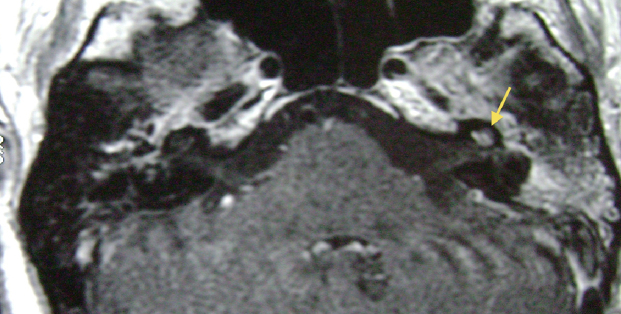
NMR weighted in T1 with gadolinium. Enhancement of contrast in the left cochlea (arrow) in a patient with Wegener's granulomatosis. *Note*: enhanced contrast in the middle ear. Patient with associated mastoiditis.

**Figure 4:**
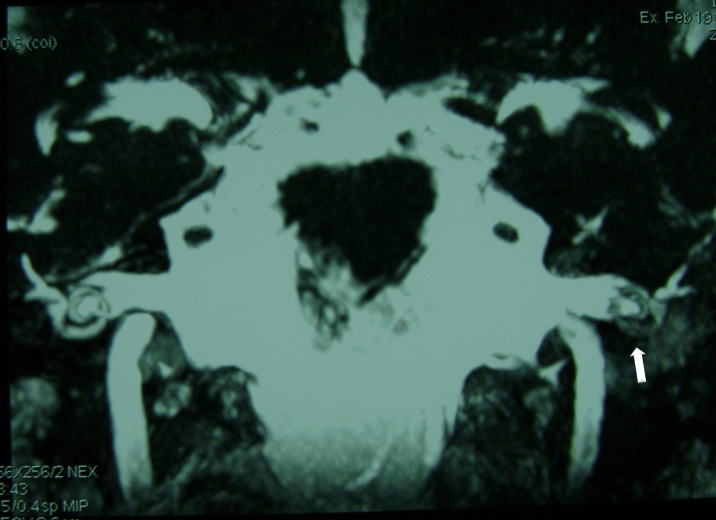
NMR FIESTA sequence, coronal view. The arrow shows hypointense signal in the cochlea and vestibular projection. This is the same patient shown in Fig. 3.

**Figure 5:**
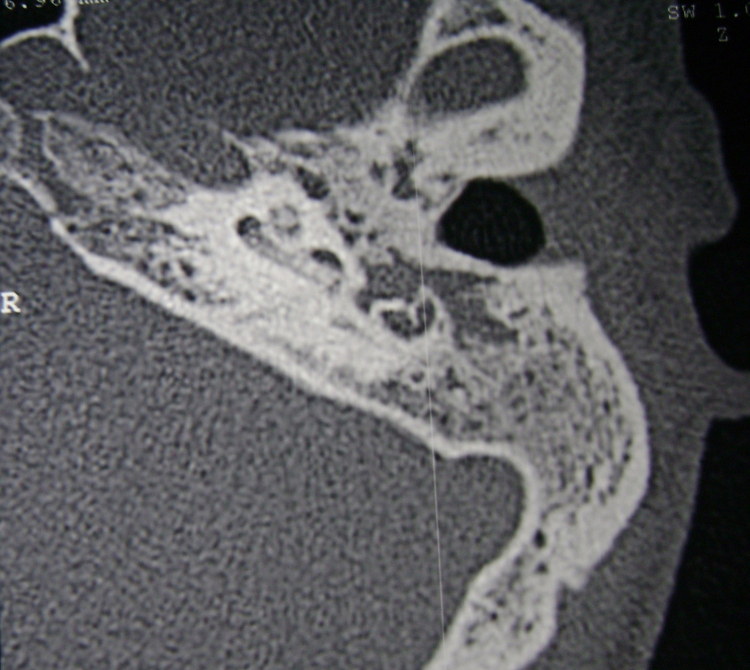
Left temporal bone CT, axial view, showing cochlear ossification. *Note*: underdeveloped mastoid filled with soft tissue-density material.

Tinnitus was reported in 14 patients (100%), vertigo in 10 patients (71%), and nystagmus in five (36%). Eight (57%) patients developed anacusis, and six (43%) progressed to mixed hearing loss (moderate to severe). In nine patients (64%), the diagnosis of labyrinthitis was confirmed with the aid of NMR: with contrast (Fig. 3) and with the FIESTA (fast imaging employing steady state acquisition) sequence (Fig. 4). The temporal bone CT scan corroborated the diagnosis in three patients (cases 2, 3 and 4) (21%) through the observation of cochlear ossification (Fig. 5).

## Discussion

It is known that the sequence of events that follow an episode of suppurative labyrinthitis typically occurs in three stages. In the acute phase, the bacteria and leukocytes appear in the perilymphatic space; in the fibrous phase, granulation tissue consisting of fibroblasts associated with neovascularization result in fibrosis, and finally, the ossification phase is characterized by metaplastic bone formation.^12^ In animals with suppurative labyrinthitis, fibrosis was observed as early as two weeks, and ossification as early as two months after infection.^8^ In the present cohort, all individuals who developed cochlear ossification were diagnosed at least six months after the infection onset, and all progressed to complete loss of hearing.

The predominant otologic diagnoses were CCOM and AOM; although the sample was small (*n* = 14), similar patterns were reported in studies with a larger number of patients.^4^, ^5^ The majority of patients with CCOM had labyrinthine fistula as an associated complication. It is assumed that the physiopathological process in these cases occurs through close contact of the infected granulation tissue below the cholesteatoma matrix with the endosteal membrane and the underlying perilymph,^1^, ^3^, ^4^, ^5^ whereas in cases of acute otitis media, it is believed that the infection extends into the labyrinth through a vulnerable or dehiscent oval window membrane.^2^, ^12^

There is little direct evidence of inflammation occurring during labyrinthitis, and the knowledge of the inflammatory response dynamics within the human inner ear is still limited.^8^ We believe that a better understanding of the inflammatory process and the possibility of defining its course in an affected individual will help to identify the type and location of the cochlear lesion, resulting in the development of customized treatments aimed at reducing the harmful effects on the delicate structures of the inner ear, thus changing the current paradigm of administering systemic corticosteroids to all cases.

Some animal studies have investigated the mechanisms of inflammation-induced inner ear disorders; however, studies in humans are limited. The reasons could be: the intralabyrinthine fluids and tissues are housed in the temporal bone complex and are difficult to collect for culture; acute hearing loss or vertigo are rarely fatal, making inner ear tissues rarely available for autopsies; the temporal bone is not usually removed in routine autopsies, thus limiting histological analysis.^1^, ^4^

Imaging tests help to overcome these obstacles and currently play an important role in diagnosis. Few medical areas have benefited from advances in diagnostic imaging as much as otology.^8^, ^9^ NMR was performed in 64% of patients in this study and it was a decisive tool to confirm the diagnosis and to assess the extent of disease.

Previous studies^8^, ^9^, ^10^, ^11^ have emphasized the importance of using gadolinium in NMR to detect inner ear inflammatory lesions. They also noted that incipient alterations in the disease process are detectable by nuclear magnetic resonance imaging. It is believed that the enhancement by contrast results from a disruption of the blood–labyrinth barrier (BLB). The BLB maintains the composition of the inner ear fluids and protects the inner ear from toxic substances through its selective permeability characteristic.^9^, ^13^

In the radiological literature, three phases of labyrinthitis are usually described: acute, fibrous stage, and labyrinthitis ossificans^14^; it is important to mention that this is a didactic division and that the concomitant occurrence of the phases is possible. In the acute phase, there is intense enhancement of the inner ear structures at T1-weighted NMR images after intravenous gadolinium injection (Fig. 3). We believe it is precisely at this stage of the disease that the NMR is essential for the diagnosis, as labyrinthitis often has unspecific symptoms and it is not possible to diagnose it by clinical means alone.

CT studies are of little help in this phase, as well as in the fibrous stage of labyrinthitis. With the replacement of intralabyrinthine fluids by fibrous tissue septa, which are characteristic of the fibrous phase, there may still be inner ear enhancement by gadolinium, although less pronounced. At the T2-weighted NMR and in the FIESTA sequence (Fig. 4) it is possible to observe the decreased signal in the inner ear. In turn, at the labyrinthitis ossificans stage, where debris and boney spicules are formed, the temporal bone CT scan (Fig. 5) is the best test to identify lesion extent and location. At the NMR there is typically no contrast enhancement, and in the FIESTA sequences and T2, hypointense signal can be observed in the labyrinthine region.

We emphasize that all patients (100%) complained of tinnitus and all of them developed some degree of sensorineural hearing loss (with or without an air-bone gap), and 71% complained of vertigo, usually correlated with infection severity. In a more comprehensive series of cases,^2^ profound or complete hearing loss was noted in all cases. That series also reported that labyrinthitis-associated vertigo was compensated in all patients. We also observed that after the acute infectious condition was over, patients with vestibular complaints showed a satisfactory recovery. Also noteworthy is the importance of taking into account the tinnitus complaint (ubiquitous in this study), which despite being quite common in other otologic diseases can indicate an incipient invasion of the cochlear neuroepithelium.

It is pertinent to observe that labyrinthitis occurred in association with other complications in all except one patient (93%); these complications usually receive greater attention due to their more urgent clinical presentation, resulting in a delay in the labyrinthitis diagnosis. When it is finally identified, the hearing loss is already established. With the current available treatment, even if it were possible to attain an early diagnosis, once the inflammatory process starts in the inner ear, it develops into an inevitable cell damage process. Unfortunately, the early diagnosis of labyrinthitis still does not change the hearing prognosis, except in cases of immune-mediated diseases, such as Wegener's granulomatosis, in which it is possible to improve hearing thresholds with specific treatment.^15^, ^16^ Concomitant complications have been widely reported in the literature,^3^, ^4^, ^5^, ^6^ which should alert otorhinolaryngologists to the importance of a careful assessment of patients with complications of otitis media, in order to rule out associated complications.

## Conclusions

Suppurative labyrinthitis was often associated with other complications and MRI helped to establish the definitive diagnosis in its acute phase. Hearing sequel of labyrinthitis was significant.

## Conflicts of interest

The authors declare no conflicts of interest.
